# Improving Access to and Delivery of Maternal Health Care Services to Prevent Postpartum Hemorrhage in Selected States in Nigeria: Human-Centered Design Study

**DOI:** 10.2196/58577

**Published:** 2025-05-07

**Authors:** Bosun Tijani, Uchenna Igbokwe, Temi Filani, Adefemi Adewemimo, Lola Ameyan, Martins Iyekekpolor, Steven Karera, Olatunji Oluyide, Emmanuela Ezike, Temidayo Akinreni, Obruche Ogefere, Victor Adetimilehin, Valentine Amasiatu, Chukwunonso Nwaokorie, Naanma Kangkum, Olufunke Fasawe, Eric Aigbogun Jr

**Affiliations:** 1 Co-creation Hub Nigeria, Lagos, Nigeria; 2 Solina Centre for International Development and Research, 8 Libreville Crescent, Wuse, Abuja, 904101, Nigeria, +234 7031135557; 3 Clinton Health Access Initiative, Abuja, Nigeria; 4 Department of Anatomy, Alex Ekwueme Federal University Ndufu-Alike, Abakiliki, Nigeria; 5 College of Medicine, Enugu State University of Science and Technology, Enugu, Nigeria

**Keywords:** human-centered design, postpartum hemorrhage, maternal health, health care services, maternal, health care, service, postpartum, facility, uterotonic, supply chain, Nigeria, interview, focus group, pregnant, female, health care workers, stakeholder, participatory, prevention, treatment, hospital setting, community

## Abstract

**Background:**

A significant cause of postpartum hemorrhage (PPH) is access to and delivery of maternal health care services. Several multisectoral strategies have been deployed to address the challenges with little success, thereby necessitating the use of human-centered design (HCD) to enhance health care delivery, particularly in PPH management.

**Objective:**

This study aims to develop facility-level solutions for optimizing uterotonic supply chain systems and health service delivery in PPH management through an HCD approach in selected Nigerian states.

**Methods:**

The research used a four-phase HCD methodology: (1) co-research, (2) co-design, (3) co-refinement, and (4) implementation. However, this paper focused on the first 3 phases. In the co-research phase, 203 interviews were conducted, involving 80 pregnant women and nursing mothers, 97 health care workers, and 26 key stakeholders. Additionally, 33 sites were observed across a 3-level continuum of care. Interviews and focus group discussions revealed insights into the distribution of health workers and observed PPH cases, alongside knowledge and administration of uterotonics. Data analysis was carried out using three key steps: (1) identifying key themes from the collected data, (2) developing insight statements that encapsulate these themes, and (3) translating each insight statement into actionable design opportunities.

**Results:**

About 150 ideas were produced and translated into 12 solution prototypes in the co-design phase. Progressive refinement following feedback from 140 stakeholders led to the selection of three final solutions: (1) implementing a referral linkage system to improve the transportation of pregnant women to nearby health facilities, (2) increasing demand for antenatal care services among pregnant women and their families, and (3) delivering a comprehensive uterotonic logistics management program for streamlined uterotonic storage and management.

**Conclusions:**

This approach aligns with global health trends advocating for HCD integration in health care programming and aims to empower local champions to drive sustainable improvements in maternal health outcomes. Judicious implementation of the developed prototypes across the states can strengthen clinical care and potentially reduce maternal health service delivery gaps.

## Introduction

Maternal mortality occurs due to complications that arise during and after pregnancy and childbirth. The major complications responsible for most maternal deaths include postpartum hemorrhage (PPH), postpartum infections, pre-eclampsia and eclampsia, complications during delivery, and unsafe abortion [1].

Globally, PPH is most pronounced in low-income countries [2], inflicting suffering on women and their families and also causing a strain on local and national health systems. Generally, PPH varies among regions, largely due to demographic and socioeconomic factors such as age, race, and social status, with low-income countries having a 12% higher occurrence [3]. One of the major drivers is limited access to timely, safe, and quality maternal care, which results in inequalities in maternal health services globally. Following a systematic review, 24.5% of maternal deaths in sub-Saharan Africa were attributed to PPH [4]. Over the past two decades, substantial efforts have been made to improve maternal care in sub-Saharan Africa, resulting in a 40% reduction in maternal mortality [5]. However, there are still significant challenges, such as substandard care, poor management skills, lack of knowledge, and delays in transferring women to the next level of care [6].

In Nigeria, PPH is the leading cause of maternal mortality, responsible for at least 21% of maternal deaths [7]. The country also bears a significant burden of maternal deaths, with 28.5% (82,000) of global maternal deaths occurring there annually [8]. Roughly 31% of female deaths in Nigeria are attributed to maternal causes, and in 2018, the country recorded a maternal mortality ratio of 512 maternal deaths per 100,000 live births [9]. Some factors contributing to this sad reality include poor access to essential medicines, including uterotonics for PPH management, and inadequate health care providers [10]. To address this threat, timely dissemination of information, primary preventive measures, comprehensive antenatal care (ANC), and strong political commitment are essential [3]. The key lies in proactive prevention, underscoring the need to analyze PPH comprehensively to develop effective mitigation and treatment strategies. Although several interventions have been carried out to prevent PPH, such as assessing coverage, acceptability, and feasibility of a comprehensive program [11-14], these interventions were health facility-centered and overlooked the broader ecosystem of care. Therefore, it was imperative to introduce the human-centered design (HCD), a collaborative method to enhance clinical care and bridge gaps in maternal health services delivery. This method involves not only health care providers but also other actors integral to providing and receiving care, including policy makers and patients [15,16].

The HCD approach has been applied successfully in global health programming, offering a human-centered approach to entrenched issues [15]. One example is the Support Sisters Intervention in the United States, a community-based peer-support intervention to help Medicaid-insured pregnant women access services and appointments [16]. In light of the global successes of using HCDs, its application to address service delivery complexities associated with maternal health care in Nigeria became imperative.

Hence, this study aimed to describe the use of HCD as an approach to develop facility-level solutions for optimizing uterotonic supply chain systems and health service delivery in managing PPH in selected Nigerian states.

## Methods

### Study Design and Setting

This study was designed as participatory research using the HCD through co-research, co-design, and co-refinement phases involving desk reviews, interviews, brainstorming, and rapid prototyping. The aim was to identify and develop facility-level solutions to strengthen the appropriate use of uterotonics for PPH prevention and treatment, reduce gaps in maternal health services, and drive improvements in uterotonic use for PPH prevention and treatment in Kano, Lagos, and Niger states in Nigeria through the development and integration of solutions to achieve a safe pre- and post-childbirth delivery experience for women in Nigeria.

This study was guided by the principle that the quality of PPH care spans the entire lifecycle of the uterotonic administration, beginning from production to proper usage within the hospital setting. It recognizes that people play a pivotal role at every stage of this process and emphasizes the importance of understanding the system to facilitate necessary improvements for better outcomes.

### HCD Approach

The data collection and synthesis involved incorporating the principles of HCD. This is a user-inclusive approach that has been adopted to address and proffer customized solutions to complex challenges that impact the public health space, as well as suit the beneficiaries of the solutions [16]. It entails a mindset that starts with the people for whom the intervention is designed and ends with new solutions that are tailor-made to suit their needs. This approach disrupts traditional methods where researchers, health care providers, and administrators design new care models based solely on studies and expert opinions, instead integrating health care users’ perspectives across all stages of the process [17-19].

**Figure 1.**
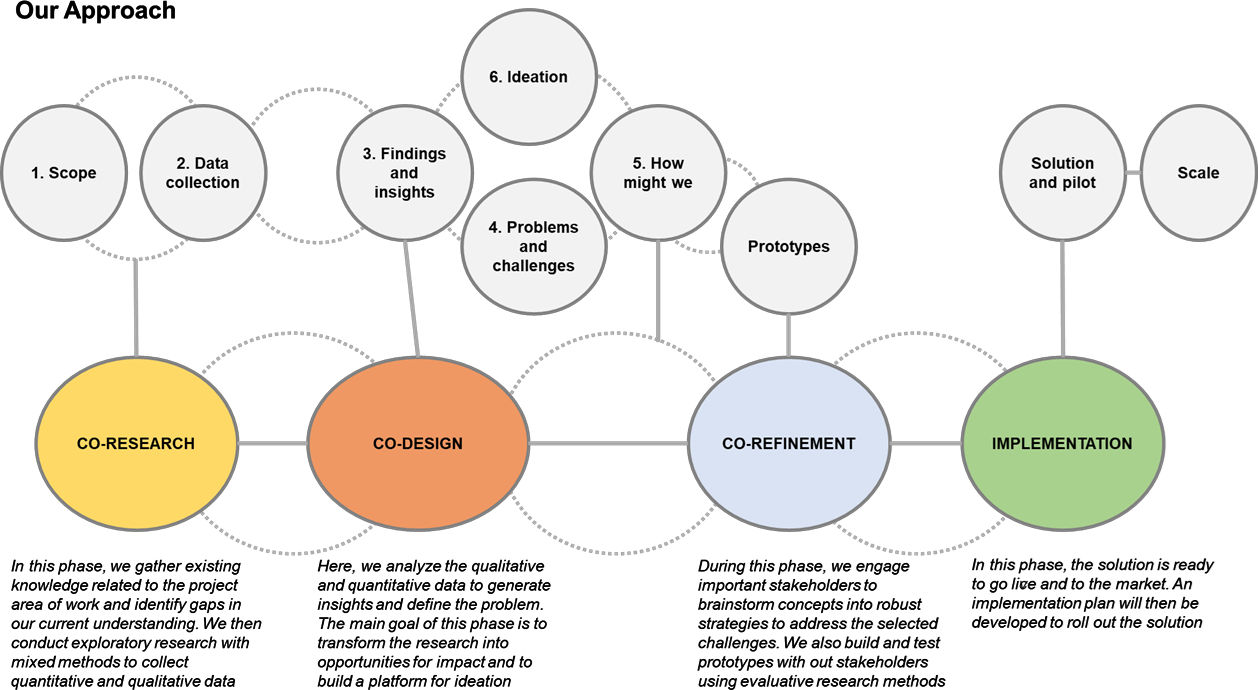
The study’s human-centered design framework.

The HCD process used in this study included co-research, co-design, and co-refinement phases (Table 1). Each of these phases enabled the discovery and an understanding of the problem and allowed us to co-create and test the developed solutions using iterative processes (Figure 1).

**Table 1.** The study’s HCD^a^ phases, approaches, and rationale.

HCD phases	Participatory approach	Rationale or comment
Co-research	Desk reviews, interviews, and site observations	Understand the PPH^b^ landscape in the states, frame research questions, design the data collection tools, and collect data
Co-design	Brainstorming and alignment	Promote creativity, idea generation, and collaborative problem-solving
Co-refinement	Rapid prototyping and streamlining (redoing, discarding, and refining)	Generate tangible results iteratively through early feedback and revision of solutions
Co-implementation	Not part of the study	Recommendations were made to the states on the need to implement the developed prototypes

a HCD: human-centered design.

b PPH: postpartum hemorrhage.

### Co-Research Phase: Understanding the User Needs, Goals and Contexts, and Tool Design (Phase 1)

The co-research phase was conducted across the 3 program states—Kano, Lagos, and Niger—from May 24 to October 24, 2022. It kick-started the HCD process and involved identifying the knowledge gaps around maternal mortality due to PPH through desk reviews to (1) understand the current status of PPH in Kano, Niger, and Lagos states; (2) frame research questions; and (3) design the data collection. The insights gleaned from this phase were subsequently translated into opportunities for impact.

### Co-Design Phase: Designing, Piloting, and Testing (Phase 2)

The co-design phase was conducted across the 3 program states from November 7, 2022, to January 10, 2023. The co-design phase aimed at transforming insights that were identified during the co-research phase into new and more structured ideas via the brainstorming process, which encouraged the champions to think expansively.

### Co-Refinement Phase: Refining the Prototypes (Phase 3)

The co-refinement phase took about 1 month to achieve between January 2 and February 23, 2023. During the co-refinement phase, each of the context-based solutions was validated through rapid prototyping.

### Data Collection

In the co-research phase, 58 champions were trained in data collection across these 3 states. Training on proper interviewing techniques and mock sessions were held across the states. This enabled the government stakeholders from the program State Ministries of Health and the State Primary Health Care Boards, assigned as HCD champions, to review existing knowledge, identify gaps, and define the research methodology needed to deepen understanding of the problem.

The methodology for data collection was selected based on the timeframe, understanding of the local context, and resources available. The following exploratory methods were selected: (1) site visit observation (live-birth observation); (2) interviews with key informants (government officials); and (3) one-on-one interviews with pregnant women, health care providers (ie, nurses, midwives, doctors, and other health professionals), traditional birth attendants (TBAs), or health facility representatives (ie, facility in-charges and administrators).

The data collection tools were developed based on identified gaps from the landscape review and to answer the research question on these core areas: facility profile, knowledge and information on the use of uterotonics, the types and challenges regarding uterotonic use, facility equipment, and uterotonic availability.

Three unique tools were developed to solicit responses from the participants: (1) an observatory tool for the site visit (health facilities); (2) key informant interview guides; and (3) survey tools for health care providers, TBAs, and pregnant women. These tools differed for each group, ensuring the questions were tailored to their various contexts.

A 3-day workshop was organized in each of the 3 program states to gather the data collected from the field activities. This workshop focused on identifying the knowledge gaps gathered, having a clear and robust understanding of the challenges to be addressed, as well as generating insights to be translated into areas for opportunities.

### Data Analysis: Synthesis and Intervention Development

The data collected (site visit observation, interviews with key government informants, and one-on-one interviews with pregnant women, health care providers, TBAs, or health facility representatives) were analyzed using a descriptive analysis method to constructively summarize data points.

Data analysis followed three steps in accordance with the HCD methodology: (1) identifying key themes from the collected data, (2) developing “insight statements” that encapsulate these themes, and (3) translating each insight statement into actionable “design opportunities” [16,20]. The synthesis and actionability of the findings from the co-research phase were carried out in the co-design and co-refinement phases.

The co-design phase featured a 2-day workshop in the program states where the HCD champions collectively brainstormed over 60 unique ideas from individual concepts and bundled them into 12 distinct robust prototypes of substance that were best suited to address the challenge. In the co-refinement phase, the solutions (prototypes) were tested with the intended users, and their feedback was integrated. This involved redoing, discarding, and refining the solutions based on a rich understanding of the context, and many potential points of failure that have been uncovered and resolved. Following the testing and feedback activity, the most viable solution was selected, one for each state, and a plan of action was created for implementation on a larger scale. These co-design and co-refinement processes were iterative to ensure the most appropriate models were achieved.

### Ethical Considerations

The study was carried out between May 24, 2022, and February 23, 2023, and was conducted in strict compliance with the ethical standards outlined in the Helsinki Declaration, ensuring the well-being of the participants. These standards encompassed obtaining informed consent, guaranteeing confidentiality, and safeguarding participants’ rights. Prior to commencement, the research received its ethical approval from the National Health Research Ethics Committee of Nigeria (NHREC) under the approval number NHREC/01/01/2007-15/03/2022 on March 15, 2022.

## Results

### Participant Groups

In total, 203 interviews were conducted among 97 health care providers, TBAs, and CHIPS (Community Health Influencers, Promoters and Services) Programme agents; 80 pregnant women or nursing mothers; and 26 government officials, with observations conducted at 33 sites across a 3-level continuum of care. Table 2 presents the participant distribution across the states.

The findings were classified based on the 3 HCD phases used in this research. Following the analysis of the data collected in the field during the co-research phase, the strong findings were subsequently categorized under common themes according to the different participant groups as described in Table 2. The findings were further synthesized into insights that were translated into opportunity areas for impact in the co-research phase.

**Table 2.** Distribution of study participants interviewed during the co-research phase.

State	Site observation (n=33), n	Doctors, nurses or midwives, TBAs^a^, and CHIPS^b^ agents interviewed (n=97), n	Pregnant women or nursing mothers interviewed (n=80), n	Government officials interviewed (n=26), n
Kano State	12	33	28	8
Niger State	11	35	26	10
Lagos State	10	29	26	8

a TBAs: traditional birth attendants.

b CHIPS: Community Health Influencers, Promoters and Services Programme.

### Co-Research: Understanding the User Needs, Goals and Contexts, and Tool Design

All of the activities in the co-research phase ensured a robust and clear understanding of the challenge to be addressed. The key insights from the co-creation phase across the 3 program states included the challenges faced by pregnant women in rural areas concerning affordability, accessibility, and awareness of health care services; health care workers’ strong commitment to maternal health despite issues with service efficiency and working conditions; observations indicating inadequacies in equipment and space management within health care facilities; and key informants expressing concerns about the current health care system’s ability to deliver adequate maternal care, emphasizing the need for ongoing improvements in service delivery and coordination.

### Co-Design: Designing, Piloting, and Testing

The workshop focused on finding solutions to the challenges from the findings from the co-research phase. These solutions aimed to provide improvements in clinical care and appropriate use of uterotonics for PPH management and treatment.

During the co-design session, ideas and concepts were synthesized, reviewed, and narrowed down to a set of concepts that could be developed for testing. The main components of this phase were framing the design challenge, generating “how might we” questions to proffer solutions to the identified challenge area, brainstorming ideas, creating appropriate storyboards for promising solutions, and conducting rapid prototyping sessions. In this phase, over 100 different ideas were submitted by champions across the various states to resolve the identified challenge statement.

### Co-Refinement: Refining the Prototypes

**Figure 2.**
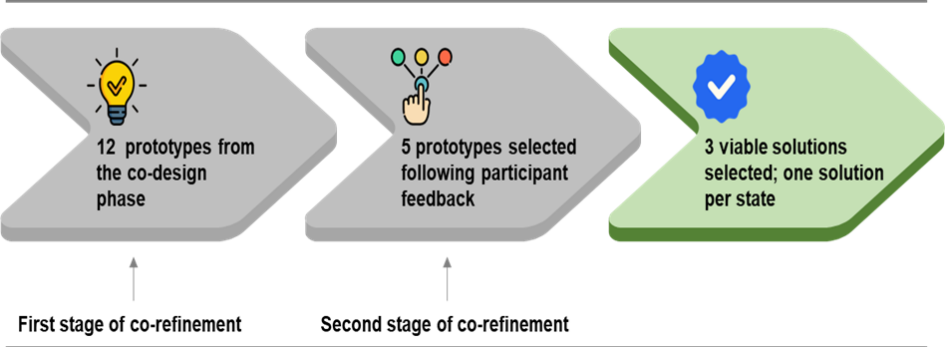
Stages of the co-refinement phase.

The co-refinement phase comprised 2 stages to contextualize the prototypes developed from the co-creation phase (Figure 2). In the first stage, 12 initial prototypes were carefully evaluated for their suitability to address the state-specific issues and narrowed down to 5 prototypes with the most promising qualities. These 5 prototypes underwent further evaluation in the second co-refinement stage to produce 3 prototypes, one for each state, that best addressed the specific issues related to optimizing uterotonic supply chain systems and health service delivery in managing PPH.

### Prototype Profile 1

#### Challenge Statement 1: How Might We Increase the Storage of Uterotonic Drugs in the Health Facilities in Kano State?

**Figure 3.**
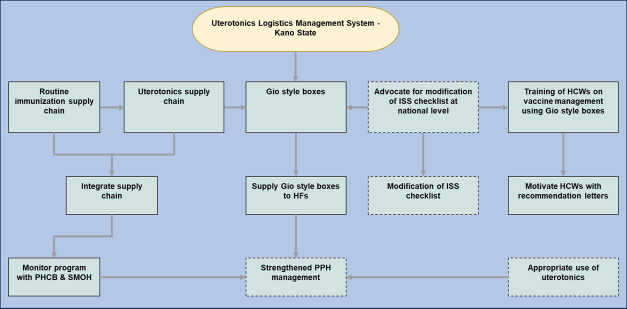
Flowchart of the Uterotonics Logistics Management System. HCW: health care worker; HF: health facility; ISS: integrated supportive supervision; PHCB: Primary Health Care Board; PPH: postpartum hemorrhage; SMOH: State Ministry of Health.

The Uterotonics Logistics Management Program is an integrative program that aims to include uterotonics in the general supply chain and storage for routine vaccinations within the state (Figure 3). This program aims to create an advocacy strategy by creating an alliance within the State Ministry of Health to include uterotonics into the integrative supply list for routine immunization.

#### Challenge Statement 2: How Might We Improve Care-Seeking Behavior for Handling PPH Cases Among Women of Childbearing Age in Kano State?

**Figure 4.**
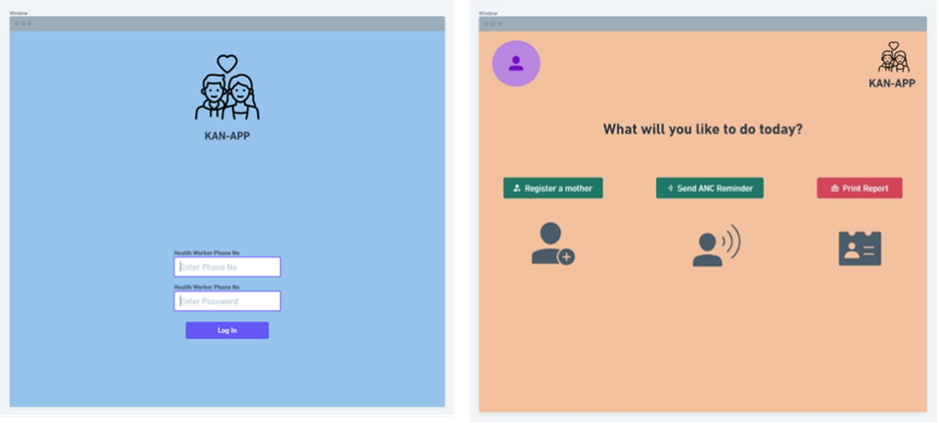
Kan-App for the increase in the demand for antenatal care services among pregnant women and their families, particularly their spouses.

The need to increase the level of awareness about PPH in Kano State is high, particularly due to women’s preference for home delivery (Figure 4), a decision that is highly influenced by their spouses. Hence, women of childbearing age may tend to demand health services like ANC following reinforcement from their husbands. The Kan-App program has three core elements for improving care-seeking behavior among women of childbearing age in Kano: (1) health care workers will be able to send reminders to husbands about the ANC visit schedules of their wives; for unmarried women or single mothers, the app will send reminders to their close relatives; (2) distribution of maternity blankets designed with key information on PPH and why women should attend ANC sessions, highlighting the role of the community in identifying the danger signs of pregnancy and the safety of facility-based delivery; and (3) radio programs to educate women on the dangerous signs of pregnancy and delivery.

### Prototype Profile 2

#### Challenge Statement 1: How Might We Improve the Transportation and Referral Linkage for the Primary Health Care Facilities?

**Figure 5.**
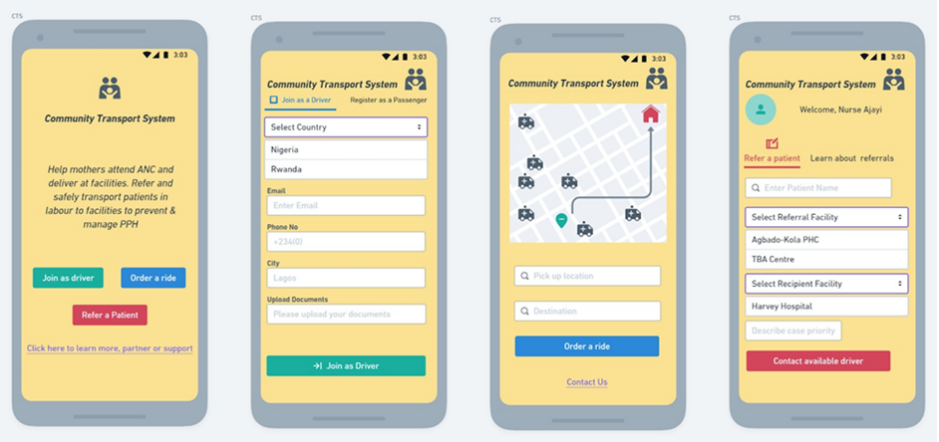
Community transport system app for improving the transportation and referral linkage for the primary health care facilities.

The community-based transport system is a simplified intervention designed to leverage the already existing network of Keke Napep drivers, Yellow Cab drivers, or the National Union of Road Transport Workers (NURTW), as well as the local security agencies in Lagos State (Figure 5). The State Primary Health Care Board in conjunction with the State Ministry of Health will develop a memorandum of understanding with the NURTW, Keke Napep, and the security agencies. A database of drivers will be developed for the 20 local government areas in Lagos State, and drivers will be informed of the MoU (memorandum of understanding) and the program, allowing them to volunteer their services and provide their contact details. Each primary health care (PHC) facility would be furnished with a list of drivers and emergency contacts, which will be shared with pregnant women as well as TBAs nearby. TBAs can use the drivers to refer women to PHC facilities. The key elements of the program include (1) pre-established communication between the health facilities, drivers, and security agencies; (2) a database of the drivers and unique communication lines; (3) unique terrain and level of care specific to hard-to-reach areas and gridlock areas (Keke Napep and Yellow Cabs); and (4) selection (volunteer), remuneration, and recognition of the drivers.

### Prototype Profile 3

#### Challenge Statement 1: How Might We Increase the Storage of Uterotonic Drugs in the Health Facilities in Niger State?

**Figure 6.**
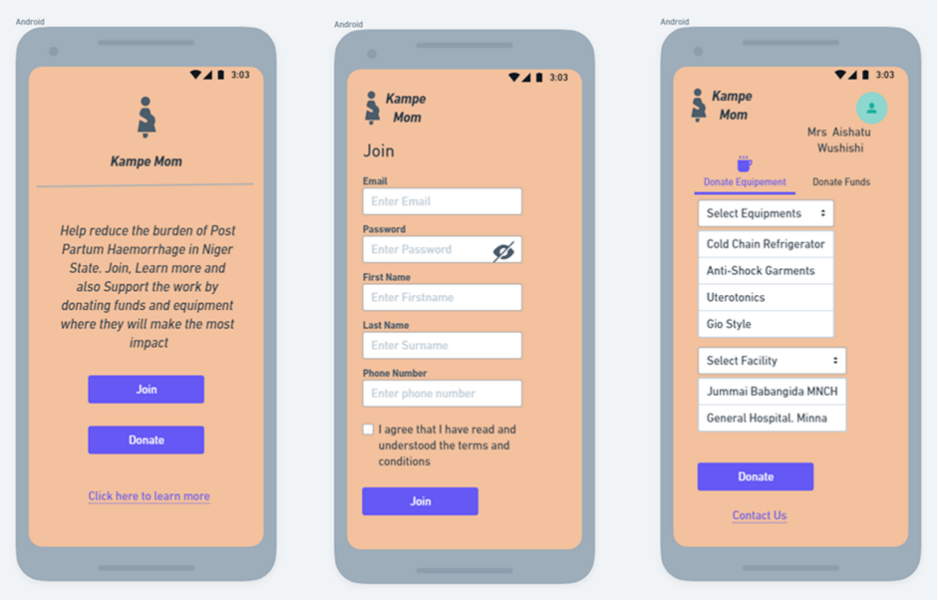
Kampe Mom app for increasing the storage of uterotonic drugs in the health facilities in Niger State.

The Niger State Primary Health Care Development Agency (NSPHCDA) Mobile and Electronic Application Platform (Kampe Mom) is an open-source web-based application that visualizes the equipment needs of PHC facilities within the state. This platform will be open to government actors (ministries, departments, and agencies), philanthropists, and organizations who are willing to contribute toward purchasing equipment for PHC facilities based on identified needs (Figure 6). The equipment will be displayed based on the order of priority and needs of the PHC facility at the time. In addition, there would be key events such as charity walks and fundraising events to create awareness of PPH and safe motherhood and generate funds that will feed into the purchase of this equipment for the PHC facilities.

The elements of the solution include the following:

- Registration of 1400 PHC facilities in Niger State on the platform. These PHC facilities will be disaggregated by location, accessibility, patient in-flow, etc.
- Equipment needed in all 25 local government areas will be captured on the platform and open to philanthropists, key decision-makers, legislators, community-based organizations, civil society organizations, women groups, etc.
- Charity walk can be placed on the platform for fundraising.
- Create awareness once a year on May 22, which is the world’s safe motherhood day. Progress updates will be provided on programs about maternal and child health.
- The target audience will be pregnant women, fathers, families, policy makers, community and religious leaders and gatekeepers, TBAs/CHIPS agents, other community volunteers, community-based organizations, civil society organizations, etc.

## Discussion

### HCD Process and Outcomes

While much of the global effort has focused on a health system–strengthening approach to reducing pregnancy-related deaths in low- and middle-income countries [21,22], less energy has been focused on solutions that take into consideration the major challenges mothers go through during pregnancy, so as to develop a client-centered solution that effectively addresses their needs. This research was aimed at proffering solutions to develop context-specific solutions to enhance service delivery for postpartum care in Lagos, Kano, and Niger states using HCD.

In the co-discovery phase, the study was able to uncover critical challenges faced by both pregnant women and health care workers in rural settings, with a focus on maternal health care access and service delivery. Pregnant women primarily struggle with affordability, accessibility, and awareness. Many of these women are housewives in rural areas, with limited financial resources and logistical difficulties in accessing health care facilities [23]. These findings are consistent with existing literature, which highlights that rural women often face significant socioeconomic barriers, including poverty, transportation challenges, and limited access to health care services [24-26].

Health care workers, on the other hand, are very committed to improving maternal health but face challenges resulting from gaps in care levels between primary and secondary facilities. The strain is particularly evident in facilities with high client volumes and limited human resources [27,28], underscoring the need for better support systems for health care providers in these settings. There were also issues of inadequate health care infrastructure, such as insufficient storage systems and overcrowded delivery wards, which could compromise the quality of care provided during critical moments like prolonged labor [28,29]. There is also the issue of the service delivery system, which requires continuous strengthening to meet the needs of these vulnerable populations. Addressing these issues will require a multifaceted approach, which should include a better system for managing the human resource problems, improved health communication strategy, demand generation, and enhanced coordination of care to ensure that both health care workers and pregnant women receive the support they need to achieve better health outcomes [11].

In this research, the co-design phase was aimed at synthesizing relevant questions to create potential solutions for the identified problems affecting the appropriate use of uterotonics and PPH care, particularly in resource-limited settings. This process highlighted the importance of involving the intended users in the HCD co-design process to ensure that the developed solutions are tailored to the specific contexts and challenges identified [30,31]. For instance, the HCD co-design phase was used to develop health solutions to enhance the comfort, health outcomes, and overall satisfaction and experience of patients [16,32].

More than 100 questions and ideas gathered from the initial co-research phase were ideated in brainstorming sessions to identify the 12 most feasible prototypes. These solutions were narrowed down by selecting the questions that most exhaustively discussed the challenges with service delivery for PPH care, suited the contexts in each state, and considered the most frequently occurring challenges and issues. This was done to ensure the final prioritized questions would generate solutions that would incite the most impact in each state. The brainstorming approach to co-design was used to further foster creative thinking and ideation, as well as generate comprehensive, user-centric solutions [16,33]. The 12 prototypes were further refined to select the most fitting solutions for the 3 states.

The implementation of brainstorming sessions in the co-design phase aided the co-refinement of the 12 prototypes. The collaborative relationships built during this session fostered the iteration and feedback process, ensuring that the prototypes selected aligned with the needs of the users and were actionable and sustainable [33,34]. The 12 prototypes were narrowed down to 3 final solutions, which were selected based on their operationalizability (how well they worked), feasibility (how possible it is to implement), and specificity (how they matched each state’s unique challenges).

### Challenges Faced and Lessons Learned

#### Stakeholder Engagement

The project team had to put mechanisms in place to consistently check in and follow up with stakeholders on aligned timelines to ensure the timely execution of activities. Moreover, additional meeting time was allocated with stakeholders for the review of data and findings to generate insights for further validation.

#### Technical Execution

Priority and preference were given to hybrid engagements or blended delivery to ensure that the entire process was worthwhile and all planned activities were completed in record time. Due to the emergence of the COVID-19 pandemic, the development of an engagement protocol for physical sessions had to be developed in accordance with globally accepted guidelines.

#### Teamwork

It became apparent that the sharing of lessons learned was instrumental to team bonding and fostered synergy within the team. The project leveraged the expertise and strengths within the consortium in cases where such was necessary.

#### Program Management

During the course of the project, appropriate and real-time communication channels and structures had to be put in place for the effective passage of information across partners and stakeholders.

#### Schedule Conflict

Some of the champions were involved in other programs that were running simultaneously as the HCD co-research workshop. This necessitated the modification of the workshop agendas to suit the needs and time demands of the participants. Going forward, the scheduling process should be improved to enable more champions to attend the workshop.

### Conclusion

The study has successfully applied HCD to develop unique solutions capable of strengthening clinical care, reducing gaps in the delivery of maternal health services, and driving improvements in the use of uterotonics for PPH prevention and treatment using statements generated from the co-research and co-design phases. The study objective of developing facility-level solutions for optimizing uterotonic supply chain systems and health service delivery in PPH management was achieved through the creation of solutions that were able to increase the demand for ANC services among pregnant women and their families, particularly their spouses, improve the storage and supply of uterotonics, and generate equitable distribution and improved capacity of health care workers to handle PPH. Although the designed solutions do not address all the issues raised, their implementation can strengthen an evidence-based approach to maternal and child health services and, in turn, improve PPH management. This implementation phase will involve deploying the uptake building of the solutions and documentation outcomes, building a comprehensive picture of the entire process, and identifying potential points for intervention.

### Study Limitations

First, this study was conducted in only 3 states within Nigeria, indicating that the developed solutions may not be applicable in other regions with different cultural, socioeconomic, and health care contexts. Second, it focused on proffering solutions to improve uterotonic supply chain systems and health service delivery in PPH management, overlooking other critical aspects like cultural and religious beliefs, as well as the quality of care. Third, the short timeframe of the study and the lack of implementation mean that the long-term outcomes or effectiveness of the solutions were not captured.

## Abbreviations

ANC: antenatal care
CHIPS: Community Health Influencers, Promoters and Services Programme
HCD: human-centered design
MDA: Ministries Departments and Agencies
MoU: memorandum of understanding
NHREC: National Health Research Ethics Committee of Nigeria
NSPHCDA: Niger State Primary Health Care Development Agency
NURTW: National Union of Road Transport Workers
PHC: primary health care
PPH: postpartum hemorrhage
SPHCDA: State Primary Health Care Development Agency
TBA: traditional birth attendant
